# Centronuclear (myotubular) myopathy

**DOI:** 10.1186/1750-1172-3-26

**Published:** 2008-09-25

**Authors:** Heinz Jungbluth, Carina Wallgren-Pettersson, Jocelyn Laporte

**Affiliations:** 1 Department of Paediatric Neurology, Neuromuscular Service, Evelina Children's Hospital, St Thomas' Hospital, Lambeth Palace Road, London, SE1 7EH, UK; 2 Clinical Neuroscience Division, King's College, London, UK; 3 Department of Medical Genetics, University of Helsinki, Helsinki, Finland; 4 The Folkhälsan Department of Medical Genetics, Helsinki, Finland; 5 Department of Neurobiology and Genetics, IGBMC (Institut de Génétique et de Biologie Moléculaire et Cellulaire), Illkirch F-67400, France; 6 Inserm, U596, Illkirch F-67400, France; 7 CNRS, UMR7104, Illkirch F-67400, France; 8 Université Louis Pasteur, Strasbourg F-67000, France; 9 Collège de France, Illkirch F-67400, France

## Abstract

Centronuclear myopathy (CNM) is an inherited neuromuscular disorder characterised by clinical features of a congenital myopathy and centrally placed nuclei on muscle biopsy.

The incidence of X-linked myotubular myopathy is estimated at 2/100000 male births but epidemiological data for other forms are not currently available.

The clinical picture is highly variable. The X-linked form usually gives rise to a severe phenotype in males presenting at birth with marked weakness and hypotonia, external ophthalmoplegia and respiratory failure. Signs of antenatal onset comprise reduced foetal movements, polyhydramnios and thinning of the ribs on chest radiographs; birth asphyxia may be the present. Affected infants are often macrosomic, with length above the 90^th ^centile and large head circumference. Testes are frequently undescended. Both autosomal-recessive (AR) and autosomal-dominant (AD) forms differ from the X-linked form regarding age at onset, severity, clinical characteristics and prognosis. In general, AD forms have a later onset and milder course than the X-linked form, and the AR form is intermediate in both respects.

Mutations in the myotubularin *(MTM1) *gene on chromosome Xq28 have been identified in the majority of patients with the X-linked recessive form, whilst AD and AR forms have been associated with mutations in the dynamin 2 *(DNM2) *gene on chromosome 19p13.2 and the amphiphysin 2 *(BIN1) *gene on chromosome 2q14, respectively. Single cases with features of CNM have been associated with mutations in the skeletal muscle ryanodine receptor *(RYR1) *and the hJUMPY *(MTMR14) *genes.

Diagnosis is based on typical histopathological findings on muscle biopsy in combination with suggestive clinical features; muscle magnetic resonance imaging may complement clinical assessment and inform genetic testing in cases with equivocal features. Genetic counselling should be offered to all patients and families in whom a diagnosis of CNM has been made.

The main differential diagnoses include congenital myotonic dystrophy and other conditions with severe neonatal hypotonia.

Management of CNM is mainly supportive, based on a multidisciplinary approach. Whereas the X-linked form due to *MTM1 *mutations is often fatal in infancy, dominant forms due to *DNM2 *mutations and some cases of the recessive *BIN1*-related form appear to be associated with an overall more favourable prognosis.

## Disease name

Centronuclear (myotubular) myopathy

## Definition

Centronuclear myopathy (CNM) is an inherited neuromuscular disorder defined by a) numerous centrally placed nuclei on muscle biopsy and b) clinical features of a congenital myopathy. Additional but inconsistent histopathological features comprise a surrounding central zone either devoid of oxidative enzyme activity or with oxidative enzyme accumulation, and, in patients with mutations in the dynamin 2 *(DNM2) *gene, radial sarcoplasmic strands surrounding the central area; signs of necrosis or excessive regeneration are usually absent in all forms of CNM.

Centronuclear myopathy exists in X-linked recessive (OMIM 310400) [1], autosomal-dominant (OMIM 160150) and autosomal-recessive forms (OMIM 255200). The term myotubular myopathy [2], introduced because of a similar appearance of affected fibres and foetal myotubes, is still used by many for the X-linked form, whilst centronuclear myopathy is a term used for both the autosomal-dominant and recessive variants of the condition.

## Epidemiology

Epidemiological data are only available for the congenital myopathies as a group but not for specific conditions. The incidence of all congenital myopathies (including central core disease, multi-minicore disease, nemaline myopathy and centronuclear myopathy) is estimated at around 0.06/1,000 live births, or one-tenth of all cases of neuromuscular disorders [3]. Regional studies in Northern Ireland [4] and Western Sweden [5], suggest a prevalence of 3.5 – 5.0/100,000 in a paediatric population. These numbers are likely to be underestimates, as histopathological expression of specific genetic defects may be variable and often non-specific, particularly at a young age.

Based on unpublished observations (JL), the incidence of molecularly confirmed myotubular myopathy in France is estimated at 2/100,000 male births per year. Whilst data regarding the overall incidence and prevalence of CNM are not available, the condition clearly occurs less frequently than central core disease and multi-minicore disease, the most common congenital myopathies, and nemaline myopathy (HJ, personal observation).

## Clinical description

Whilst creatine kinase (CK) is normal or only slightly elevated in all forms of centronuclear (myotubular) myopathy, the clinical picture is highly variable depending on the causative mutation.

**Figure 1:**
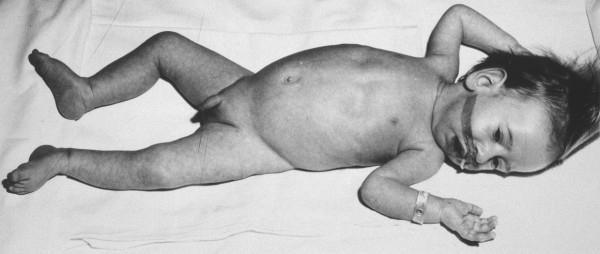
**Male infant with X-linked centronuclear ("myotubular") myopathy due to a mutation in the myotubularin *(MTM1) *gene. **Note generalised hypotonia and myopathic facial appearance with elongated face and inverted V-shaped mouth. (Reproduced from MedLink^®^Neurology, with permission)

The X-linked form due to mutations in the myotubularin *(MTM1) *gene has been clinically well characterised and usually gives rise to a severe phenotype in males presenting at birth with marked weakness and hypotonia, external ophthalmoplegia and respiratory failure (Figure 1) [6-19]; a preceding family history of either male neonatal deaths or miscarriages is common. Signs of antenatal onset comprise reduced foetal movements, polyhydramnios and thinning of the ribs on chest radiographs [20,21] and are only rarely observed in other congenital myopathies. Birth asphyxia may be the presenting feature [10,22]. Affected infants are often macrosomic, and length above the 90^th ^centile and large head circumference may serve as a diagnostic clue [23,24]. Testes are frequently undescended [25]. In the majority of cases the course is fatal within the first months of life, but a proportion of affected males may survive into their teens or beyond [19,26,27]. Although a small proportion of boys may be only mildly affected in the neonatal period and thereafter [6,18,19,22,27-29], in the majority of long-term survivors survival depends on a substantial degree of medical intervention and often constant ventilation. A range of medical complications in some long-term survivors comprising pyloric stenosis and cavernous haemangiomas of the liver [26] has also been reported and may indicate wider expression of the defective protein. Additional genital abnormalities have been described in affected males with contiguous gene syndromes [30], and is due to loss of the adjacent MAMLD1 *(Cxorf6) *gene [31].

The majority of carriers of the X-linked formare asymptomatic but a few may show signs of mild muscle weakness [10,32-34]. Presentation may be overt in females, especially if additional genetic abnormalities such as skewed X-inactivation [34-39] or structural X-chromosomal abnormalities are present [40]. Urinary incontinence, primary or secondary, may be an additional feature indicating smooth muscle involvement [33,37].

Both autosomal-recessive and autosomal-dominant forms have been well documented and differ from the X-linked form regarding age at onset, severity, clinical characteristics and prognosis [18,41]. As a general rule, autosomal-dominant forms have a later onset and milder course than the X-linked form, and the autosomal-recessive form is intermediate in both respects, but these differences are quantitative rather than qualitative. Most reports concerning autosomal-recessive and dominant forms of CNM predate the molecular resolution of these conditions and are likely to reflect genetically heterogeneous conditions; however, the recent identification of the genes implicated in subgroups of recessive and dominant CNM offers the prospect of more precise genotype-phenotype correlative studies in future.

The autosomal-recessive form [10,41-52] is characterised by facial weakness including severe involvement of the masticatory muscles [53], and ocular abnormalities such as ptosis and external ophthalmoplegia. A recent French series distinguishes early and late onset forms with or without ophthalmoplegia; it remains to be seen if those distinctions are reflective of underlying genetic heterogeneity [50]. Weakness is usually prominent proximally but there may be additional distal weakness and wasting in the lower limbs, and foot abnormalities are frequently found [54]. Other skeletal deformities including high arched palate and scoliosis are common [55]. Respiratory involvement may be severe [52], and an associated cardiomyopathy has been documented in a few recurrent and sporadic cases [44,56,57]. As in carriers of the X-linked form, urinary incontinence may be an associated feature [58]. In the absence of severe cardiorespiratory involvement, the prognosis appears favourable. Whilst most of the features of supposedly recessive cases of centronuclear myopathy were reported in genetically unresolved cases, identification of homozygous recessive mutations in the amphiphysin 2 (*BIN1) *gene in 4 patients form 3 families [59] allows initial genotype-phenotype correlations, although, considering the small number of cases, the range of clinical features associated with mutations in this gene is likely to expand further in the future. Clinical features in the patients identified to date suggested a phenotype of intermediate severity between the X-linked recessive and the dominant forms, with onset from birth to childhood and mild progressive proximal weakness but no respiratory impairment severe enough to require ventilatory assistance. Cardiac involvement was not present in these patients but, notably, appears to be a feature in the *BIN1 *knockout mouse [60].

Most patients with the autosomal-dominant form of CNM are more mildly affected than those with the X-linked or autosomal-recessive forms with a widely variable age of onset [18,61-75]. The distribution of weakness is predominantly proximal with additional distal involvement, external ophthalmoplegia and ptosis; in some cases, prominent calf muscle hypertrophy may be an additional feature [50].

The autosomal-dominant form of CNM due to mutations in the dynamin 2 *(DNM2) *gene may be of variable severity depending on the part of the protein affected. Dominant *DNM2 *mutations affecting the dynamin 2 middle domain reported to date appear to be associated with a mild clinical phenotype characterised by normal early motor developmental milestones, onset in adolescence and a slowly progressive course with loss of independent ambulation uncommon before the 6th decade [76-78]. In addition to signs of proximal weakness, exercise-induced myalgia may be a presenting feature. Ocular involvement, particularly ptosis, is almost invariable and distal muscle involvement, particularly in the lower limb, may precede more proximal weakness; the latter finding corresponds to a sequential pattern with early involvement of the ankle plantarflexors, namely the medial gastrocnemius, followed by signal changes in the posterior and, eventually, anterior compartment of the thighs [77,78]. Contractures other than those affecting the Achilles tendon and/or long finger flexors are rare. Electromyogram (EMG) and nerve conduction studies may show mild signs of axonal peripheral nerve involvement in addition to prominent myopathic changes [77,79]. Whilst dominant mutations affecting the dynamin 2 middle domain have been associated with a mild phenotype of CNM, a more severe presentation with neonatal onset has been recently attributed to heterozygous *de novo *dominant mutations affecting the pleckstrin homology (PH) domain of the dynamin 2 protein, a protein domain also altered in the CMTDIB neuropathy [80,81]. Like other patients with CNM, these had marked ocular involvement including ptosis and ophthalmoparesis, and, despite a severe and early presentation, gradually improved over time. However, whilst cardiorespiratory function in *DNM2*-related CNM has been normal in most reported cases those with early onset may develop restrictive respiratory impairment over time [81]. Electrophysiology showed exclusive myopathic but no neuropathic changes.

Centronuclear myopathy due to a heterozygous *de novo *dominant mutation in the skeletal muscle ryanodine receptor *(RYR1) *gene has to date been reported in only one case [82], with clinical features comprising extraocular involvement, generalized weakness, moderate bulbar and respiratory impairment similar to multi-minicore disease (MmD), due to recessive mutations in the *RYR1 *gene [83,84]. Although the frequency of *RYR1 *mutations in CNM is currently uncertain, malignant hyperthermia susceptibility reported in a case of CNM in the premolecular era [85] may indicate more widespread *RYR1 *involvement in CNM.

## Aetiology

Centronuclear (myotubular) myopathy exists in X-linked, autosomal-recessive and autosomal-dominant forms.

The X-linked recessive form ("myotubular myopathy") has been genetically well characterised. Following initial linkage studies and assignment of a locus to chromosome Xq28 [40,86-94] mutations in the myotubularin *(MTM1) *gene have now been identified in more than 90% of affected males [19,95-100]; molecular genetic analysis of the *MTM1 *gene is now widely available as a routine diagnostic service [34,101,102]. Disease-causing sequence changes include deletions/insertions, nonsense, missense and splice mutations (approximately 25% each) [97,102]. Three substitutions account for 15% of all *MTM1 *mutations; these are the splice mutation c.1261-10A>G (intronic, upstream of exon 12) resulting in the insertion of three amino acids FIQ at position 420 (7.3%), R241C encoded by exon 9 (4%), and c.141-144 delAGAA resulting in a frameshift at amino acid 48 in exon 4 (4%) [28]. Other mutations have been reported in a few families or are unique. *MTM1 *mutations are distributed throughout the entire coding sequence, but localise most frequently (in descending order) to exons 12, 4, 11, 8 and 9 [97-99,102-107]. Maternal carrier state of *MTM1 *mutations is estimated at 85% and is thus more common than statistically expected for a severe X-linked disease [97,102]; maternal mosaicism has been reported in a few families [97,108,109] with important implications for genetic counselling regarding future pregnancies.

Genotype-phenotype correlative studies have been difficult because many mutations are private to individual families and clinical severity associated with specific mutations may vary even within the same families; however, one large series demonstrated that, while most mutations are associated with the severe phenotype, some non-truncating mutations outside of the catalytic domain may carry a more favourable prognosis [19,27,28,110].

Screening of the *MTM1 *gene should be considered in females with suggestive clinical and histopathological features; although usually asymptomatic or only mildly affected, carriers may manifest severe symptoms in the presence of skewed X-inactivation and/or structural alterations involving the X-chromosome such as interstitial deletions [34-40].

Myotubularin belongs to the large family of dual-specificity phosphatases, playing a role in the epigenetic regulation of signalling pathways involved in growth and differentiation; mutations in some human myotubularin homologues have been associated with two specific forms of peripheral neuropathies of the Charcot-Marie-Tooth (CMT) type, CMT 4B1 [111,112] and CMT 4B2 [113,114]. A specific function has been proposed for myotubularin in dephosphorylating phosphatidylinositol 3-phosphate [PtdIns3*P*] and PtdIns(3,5)P. These two phospholipids are second messengers with a crucial role in membrane trafficking; by dephosphorylation of PtdIns(3,5)P2, myotubularin also produces PtdIns*5P*, whose function is not fully characterised [95,115-126]. In addition to the catalytic site, myotubularins form homo- and heterodimers and contain lipid and protein binding sites; these domains include a GRAM-PH, a coiled-coil region and a putative PDZ binding site. Concerning myotubularin, no protein interactors have been characterised to date in skeletal muscle. The deleterious effect of specific *MTM1 *mutations may be due to either destabilisation of the 3-D structure or loss of enzymatic activity, although it is possible that a few mutations affect existing but not yet identified protein-protein interactions in muscle.

Observations in an *MTM1*-related mouse model [127] suggest a role of myotubularin in muscle fibre maintenance but not in myogenesis. A recent gene expression profiling study in muscle harbouring *MTM1 *mutations revealed upregulation of transcripts for cytoskeletal and extracellular matrix proteins within or around atrophic myofibres, indicating that remodelling of cytoskeletal and extracellular architecture plays a role in the atrophy and intracellular disorganization observed in X-linked myotubular myopathy [128]. Prolonged expression but eventual decrease of developmentally regulated proteins in muscle from affected infants (see also paragraph on Diagnostic methods below) suggests maturational delay rather than complete developmental arrest in this condition.

Dominant forms of centronuclear myopathy have been associated with mutations in two genes, the dynamin 2 *(DNM2) *gene on chromosome 19p13.2 [76] also implicated in dominant intermediate (CMTDIB) [80] and axonal (CMT2) [129,130] forms of Charcot-Marie-Tooth disease, and the skeletal muscle ryanodine receptor *(RYR1) *gene on chromosome 19q13.1 in one isolated case [82].

The *DNM2 *gene consists of 22 exons [80] and encodes a large GTPase protein involved in actin cytoskeleton assembly [131] and centrosome cohesion [132]. In addition, DNM2 is implicated in membrane trafficking from the plasma membrane and Golgi, to allow the formation and fission of budding vesicles [133]. It is of interest to note that myotubularin, implicated in the X-linked form of CNM, has also been implicated in membrane trafficking and endocytosis, although its precise function remains to be determined. Recent studies on cells transfected with CNM-related *DNM2 *mutants suggest lack of localisation to the centrosome and centrosome malfunction as a possible pathogenetic mechanism in *DNM2*-related CNM. However, the impacts of mutations on allosteric enzymatic activity and membrane remodelling properties of dynamin 2 remain to be investigated. Recurrent and *de novo DNM2 *mutations were originally identified following a positional candidate approach in 11 families with a mild form of autosomal-dominant centronuclear myopathy [76]. The most common mutation is a 1393C>T change found in 6 unrelated families resulting in an arginine to tryptophane substitution at position 465. Dynamins are structurally complex proteins composed of 5 different domains; CNM-causing mutations identified to date mainly localise to the middle domain involved in protein self assembly and centrosome localisation [76], whereas those associated with CMTDIB have been identified within the pleckstrin homology (PH) domain [80]. More recently, heterozygous *de novo *dominant *DNM2 *mutations affecting the PH domain have also been identified in a more severe CNM phenotype without any early peripheral nerve involvement and characterised by neonatal onset but gradual improvement over time [81]. The potential overlap between myogenic and neurogenic findings in families with mutations in this region is currently being explored [77,79].

Features of centronuclear myopathy associated with the skeletal muscle ryanodine receptor *(RYR1) *gene have to date only been reported in one single case with a *de novo *dominant mutation resulting in a serine to leucine substitution at position 4112 [82]; the *RYR1 *gene had been considered as a candidate in this patient because of the frequent observation of multiple central nuclei in other *RYR1*-related phenotypes and the suggestion of a clinical continuum and overlap of radiological features on muscle MRI. Functional studies on patient-derived, MyoD-transformed fibroblasts indicated that cells harbouring this mutation may be hypersensitive to depolarization, but it remains unclear how the change gives rise to the appearance of CNM. The frequency of the *RYR1*-related form of CNM is currently uncertain.

Mutations in the amphiphysin-2 *(BIN1) *gene on chromosome 2q14 have been recently identified in a small proportion of cases with the recessive form of centronuclear myopathy [59] but further genetic heterogeneity is expected. The *BIN1 *gene is organised in 20 exons, the protein exists in at least 10 different isoforms subject to alternative splicing and, in addition to muscle, the gene is expressed in a number of different tissues including central and peripheral nervous systems [134,135]. *BIN1 *was considered a candidate for genetically unresolved forms of CNM because of functional characteristics shared with other CNM-associated genes, namely a phosphoinositide-regulated role in membrane modelling [136], and the presence of a muscle phenotype in the Drosophila melanogaster mutant [137]. The amphiphysin 2 muscle-specific isoform features an N-terminal amphipathic helix thought to be involved in creating membrane curvature, a BAR (Bin1, Amphiphysin, RVS167) domain that homodimerizes and maintains the curvature, a phosphoinositide-binding domain, and an SH3 domain interacting with dynamin 2 and other proteins [136,138]. Functional studies on the three mutations identified to date suggest that *BIN1 *missense mutations in the N-BAR domain affect membrane curvature, whilst a truncating mutation in the SH3 domain appears to abolish amphiphysin2-dynamin 2 interactions [59]; this indicates the importance of amphiphysin 2-dynamin 2 coupling for normal muscle function and suggests a possible alteration of T-tubule organisation, as amphiphysin 2 was previously proposed to have a role in this process [137,139].

In addition to the *BIN1*-related form, heterozygous missense variants in hJUMPY, a novel phosphoinositide phosphatase with functional similarities to myotubularin, were recently identified in two sporadic cases with features of centronuclear myopathy and an additional *DNM2 *mutation in one case [140]. These variants were shown to decrease the enzymatic activity of hJUMPY in *in vitro *and *in cellulo *experiments. It is unclear whether the phenotype in those cases is due to digeny or recessive inheritance with an undetected second mutation, and clarification of the implication of hJUMPY awaits the characterisation of additional patients with mutations in this gene.

Genes implicated in various forms of centronuclear myopathy have been summarised in Table 1.

**Table 1:** Genes implicated in X-linked recessive, autosomal-recessive and autosomal-dominant centronuclear myopathy.

**Gene**	**Gene product**	**Locus**	**Inheritance**	**Reference**
*MTM1*	Myotubularin	Xq28	Recessive	Laporte et al. (1996) [96]
*DNM2*	Dynamin 2	19p13.2	Autosomal-dominant	Bitoun et al. (2005) [76]
RYR1*	Skeletal muscle ryanodine receptor	19q13.1	Autosomal-dominant	Jungbluth et al. (2007) [82]
*BIN1*	Amphiphysin 2	2q14	Autosomal-recessive	Nicot et al. (2007) [59]
MTMR14**	Myotubularin-related protein 14 (hJUMPY)	3p25.3	uncertain	Tosch et al. (2006) [140]

*A mutation in the *RYR1 *gene has only been identified in one isolated case to date.

**Mutations in the *MTMR14 *gene have been identified in only two families with uncertain mode of inheritance.

## Diagnostic methods

The diagnosis of CNM depends on the presence of typical histopathological findings on muscle biopsy in combination with suggestive clinical features; muscle MR imaging may complement clinical assessment and inform genetic testing in cases with equivocal features.

**Figure 2:**
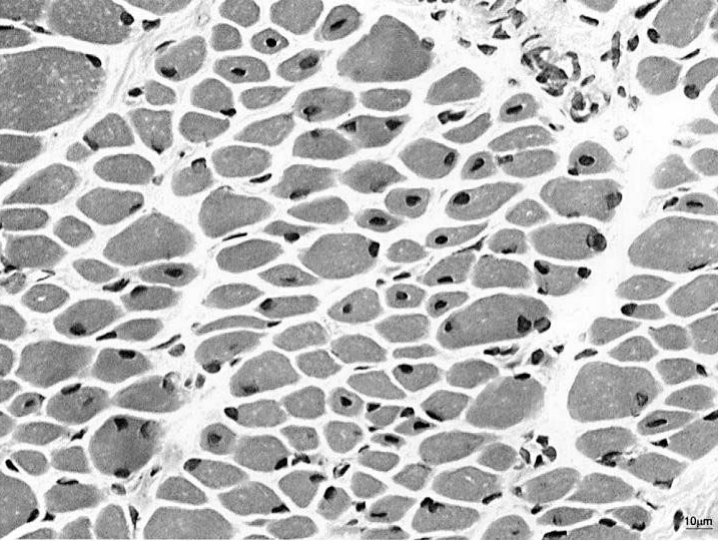
**Muscle biopsy from the quadriceps taken at 3 months of age from a girl with X-linked centronuclear ("myotubular") myopathy due to a mutation in the myotubularin *(MTM1) *gene and extremely skewed X-inactivation, H&E stain, transverse section.** Note marked variability in fibre size, moderate increase in connective tissue and numerous central nuclei.

On muscle biopsy, centronuclear (myotubular) myopathy is characterised by centrally placed nuclei surrounded by a perinuclear halo devoid of myofilaments [2] and occupied by mitochondrial and glycogen aggregates (Figure 2) The characteristic central nuclei are seen in all muscles, including extra-ocular muscles [141], and may affect up to 90% of fibres [52]. Some autosomal cases of centronuclear myopathy may also feature a radial arrangement of sarcoplasmic strands on NADH staining [50]; it currently appears that this is a feature in most cases of centronuclear myopathy caused by mutations in the *DNM2 *gene [76]: Whilst radial arrangement of sarcoplasmic strands appears to be common in mild forms of *DNM2*-related CNM due to mutations affecting the middle domain [77], this finding appears not to be as prominent in more severe and early presentations due to mutations affecting the pleckstrin homology (PH) domain, or in other genetically distinct forms of CNM. Type 1 predominance and hypotrophy [14,142] are commonly associated features, and may precede the appearance of internal nuclei [143]; there may be compensatory type 2 hypertrophy in a small number of fibres [25], and a deficiency of type 2B fibres with relative increase in undifferentiated type 2C fibres [142].

More recently, Pierson *et al*. [110] were able to correlate *MTM1 *mutation type and pathologic findings and could demonstrate that missense mutations are associated with increased myofibre diameter compared to nonsense mutations.

Histopathological changes may progress over time and marked increases in fat [144] and connective tissue [37,78,145] can at times be a striking feature. Associated core-like structures have occasionally been reported [47,144] and may be associated with mutations in both the skeletal muscle ryanodine receptor *(RYR1) *gene [82] and the *DNM2 *gene [78].

Reported histopathological findings in carriers of the X-linked form range from normal appearance in clinically asymptomatic mothers [11] to findings similar to those in affected males, as reported in a female with the full clinical picture of myotubular myopathy due to skewed X-inactivation [37].

On electron microscopy (EM), immaturity of neuromuscular junctions and junctional changes comprising reduction of acetylcholine receptors on immunoperoxidase stains [141] and simplification of the postsynaptic membrane with paucity of secondary synaptic clefts [146] have been reported but the molecular basis for this observation remains uncertain. The great majority of CNM-related electron microscopy studies either predate the molecular resolution of these conditions or concern X-linked myotubular myopathy, and there are currently not sufficient data for more detailed EM genotype-phenotype correlative studies with a view to the more recently identified genes.

Immunohistochemical studies in CNM are mainly available for the X-linked form and have demonstrated consistent but non-specific abnormalities: persistent foetal expression pattern of various proteins including the cell surface protein N-CAM [146], myosin [32,147], vimentin and desmin [145,148,149] have been reported in male infants with the X-linked forms, but more recent immunohistochemical studies on sequential biopsies in long-term survivors [150] suggest that the expression of developmentally regulated proteins eventually decreases as in healthy individuals. Other proteins abnormally expressed in myotubular myopathy include laminin and collagen components [145].

**Figure 3:**
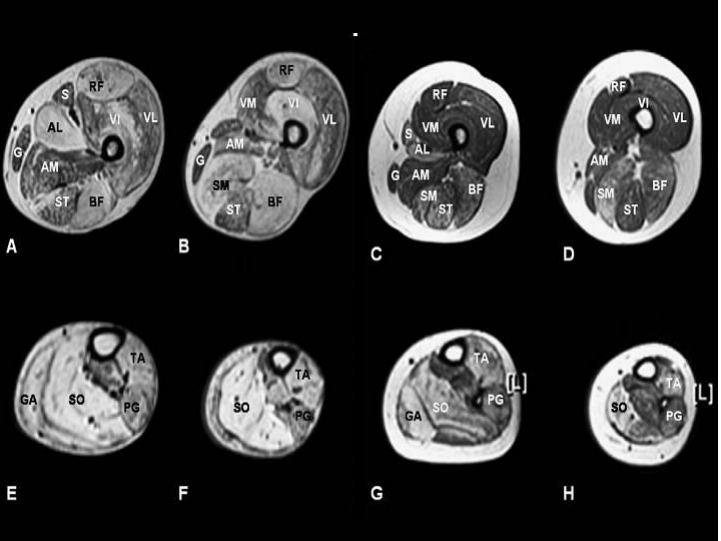
**Selective muscle involvement in a 59-year-old man (A, B, E, F) and his 28-year-old daughter with centronuclear myopathy (C, D, G, H) due to a mutation in the dynamin 2 *(DNM2) *gene, muscle MRI, transverse, T1-weighted sections from the proximal (A, C) and distal (B, D) thigh and the proximal (E, G) and distal lower leg (F, H). **In the thigh there is increased signal intensity within the adductor longus (AL), semimembranosus (SM), rectus femoris (RF), biceps femoris (BF), and vastus intermedius (VI) muscles with relative sparing of the adductor magnus (AM), gracilis (G), sartorius (S), semitendinosus (ST), vastus lateralis (VL), and vastus medialis (VM) muscles. Within the lower leg, there is predominant involvement of the gastrocnemius (GA), soleus (SO) an tibialis anterior (TA) muscles with relative sparing of the peroneal group (PG). Muscle involvement, particularly within the thigh, is milder in the daughter compared to her father. The pattern is distinct from that reported in congenital myopathies associated with mutations in the skeletal muscle ryanodine *(RYR1) *gene. (Figure courtesy of Dr Carsten Boennemann and Dr Joachim Schessl, reproduced from Schessl et al., Neuromuscular Disorders 2007; 17:28–32, with permission from Elsevier).

Muscle MRI findings have been reported in autosomal forms of CNM due to mutations in the *DNM2 *[77,78] and the *RYR1 *[82] genes. Muscle MRI in cases of centronuclear myopathy secondary to mutations in the *DNM2 *gene show a characteristic progressive sequence (Figure 3) with early involvement of the ankle plantarflexors and subsequent signal changes within the hamstring muscles and, finally, the anterior thigh. This sequence and also the prominent adductor longus and rectus femoris involvement reported in one family [78] is distinct from cases with mutations in the *RYR1 *gene [82,151] and may guide genetic testing in autosomal cases, particularly as cores on oxidative stains may be an additional finding in both *DNM2- *and *RYR1-*related forms [78,82]. Muscle imaging findings in *MTM1*-related CNM have only been reported in one manifesting female carrier [152] and are currently not documented for the recessive form due to mutations in the amphiphysin2 *(BIN1) *gene.

DNA sequencing of the exons and exon-intron boundaries of the implicated genes is used to confirm the diagnosis at the molecular level. For *MTM1 *sequencing, it is recommendable to start by investigating the more frequently implicated exons although mutations have been found distributed throughout the gene. With regard to *DNM2 *mutation screening, mutations identified to data are clearly concentrated in the middle and PH domains and these hotspots should be checked first. Due to lack of clear genotype-phenotype correlations and in cases where the genetic segregation of the disease cannot be assessed unambiguously, it is advisable to examine all three genes (*MTM1*, *BIN1 *and *DNM2*) in the search for molecular confirmation of the diagnosis.

RNA sequencing may be used if tissue or cultured cells are available from the patient, as the implicated genes appear to be ubiquitously expressed. For the X-linked form, the vast majority of the known *MTM1 *mutations lead to a decrease in protein levels in cultured myoblasts, fibroblasts or lymphoblastoid cell lines [153]; based on only a few cases studied, mutations in the *BIN1 *and *DNM2 *gene do not seem to affect protein levels in such cell types. In addition, investigation of the RNA integrity or protein levels, although not used routinely, might reveal mutations in introns or regulatory sequences that remain undetected by DNA sequencing [100].

## Differential diagnosis

Central nuclei on muscle biopsy are not pathognomonic, and other neuromuscular disorders with a secondary increase in internal nuclei have to be considered in the differential diagnosis of CNM. Congenital myotonic dystrophy is a histopathological phenocopy [154] of the X-linked form, myotubular myopathy, and ought to be considered and excluded in the first instance by obtaining a detailed family history, clinical examination of the mother and specific genetic testing as indicated, before embarking on a muscle biopsy. In addition, although often already unlikely on clinical grounds, other causes of severe neonatal hypotonia ought occasionally to be excluded by more specific testing, including the other congenital myopathies, the congenital muscular dystrophies, spinal muscular atrophy, myasthenic disorders and motor neuropathies.

The autosomal-dominant form of myotubular myopathy [50,61-63,76] also has to be differentiated from myotonic dystrophy and other autosomal-dominant disorders with numerous central nuclei on muscle biopsy, particularly in cases where mutations in the currently known CNM genes have been excluded, as clinical findings such as cataracts or electrical myotonia (*i.e*. myotonic bursts on needle EMG) [155,156] suggest that some of the families reported in the premolecular era were affected by myotonic dystrophy rather than autosomal-dominant centronuclear myopathy. A facioscapulohumeral distribution of weakness in other families [45,75] should lead to consideration of facioscapulohumeral muscular dystrophy in the differential diagnosis of autosomal-dominant centronuclear myopathy.

## Management

No curative treatment is currently available for any form of CNM and management is essentially supportive, based on a multidisciplinary approach.

X-linked myotubular myopathy is the most severe form of CNM and usually, but not invariably, follows a fatal course over days and weeks. Occasionally, long-term survival has been reported but often depends on the degree of respiratory intervention; a few male infants may be more mildly affected from the outset with better long-term prognosis [6,18,19,22,27-29]. The decision regarding the duration of respiratory support is not an easy one, but as no firm prognostic criteria have yet been established [19], an at least initially proactive stance should be taken and any respiratory management decision should be made on an individual basis rather than on diagnosis alone. The latter approach is particularly advisable in cases with neonatal presentation where the X-linked form has been excluded, as recently described patients with recessive mutations in the amphiphysin 2 *(BIN1) *[59] gene and dominant mutations in the dynamin 2 *(DNM2) *gene [76] follow a milder course and may even improve over time. Patients who survive beyond the neonatal period without immediate ventilatory requirement will need close monitoring of their respiratory function, including polysomnography studies where needed, and are likely to benefit from initiation of non-invasive nocturnal ventilation as indicated [157]. Follow-up care of those on nighttime ventilation should include regular cardiac assessments considering the risk of associated cor pulmonale [158,159]. Respiratory infections should be treated actively.

Feeding difficulties usually feature in infants with X-linked myotubular myopathy and may occur also in patients with severe recessive and dominant forms of CNM, requiring input from a speech therapist who may also promote normal speech if dysarthria is present.

As in other congenital myopathies, regular physiotherapy is aimed at the preservation of muscle power and function and the prevention of contractures; considering often prominent axial involvement, exercises promoting endurance and truncal stability such as swimming and riding [160] may be particularly useful. If orthopaedic complications evolve in the course of the disease, those may be managed surgically where conservative approaches have failed, and only at centres with experience in the management of neuromuscular disorders. As in other neuromuscular conditions, post-operative mobilisation ought to be rapid in order to avoid adverse effects of prolonged immobilisation such as muscle atrophy. In the most severe cases where walking can not be achieved without additional support, independent ambulation may be promoted by appropriate rehabilitative measures such as provision of weight-bearing calipers.

Malignant hyperthermia, an abnormal response to muscle relaxants such as succinylcholine and volatile anaesthetics [161,162], has been previously only reported in one genetically unresolved case of CNM [85] and may reflect the recently documented involvement of the *RYR1 *gene in the condition [82]. As malignant hyperthermia is not a recognised feature of other genetically determined forms of CNM, this complication ought to be mainly anticipated in genetically unresolved cases or those due to *RYR1 *mutations, although a cautious approach is generally advisable for patients with muscle disorders considered for general anaesthesia.

## Genetic counselling

Genetic counselling should be offered to all patients and families in whom a diagnosis of CNM has been made. Only the identification of the causative mutation will determine the mode of inheritance in each individual family. In families with mutations in the myotubularin gene *(MTM1)*, it is important to note that some women, who do not show in their lymphocyte-derived DNA the mutation identified in their affected son, may still carry a risk of recurrence because of germinal mosaicism for the mutation [97,108,109]. Mutational analysis of *MTM1 *is now available as a diagnostic service, and in future the same might apply to screening of the more recently identified dynamin 2 *(DNM2) *and amphiphysin 2 *(BIN1) *genes associated with dominant and recessive forms of CNM, currently mainly available on a research basis. Considering the possible important prognostic implications depending on the underlying genetic defect, there is clearly a need for the establishment of a wider diagnostic network for CNM.

## Prognosis

The prognosis of CNM is vaguely related to the mode of inheritance (see paragraph on Clinical description) with the X-linked form being more severe than dominant or recessive forms, respectively; whilst the mortality of X-linked myotubular myopathy is very high in infancy, some rare cases who are usually milder from the outset may achieve a reasonable quality of life [27,28].

As only few genetically resolved families with dominant and recessive forms of CNM due to mutations in the *DNM2 *and *BIN1 *genes have been reported to date, genotype-phenotype correlations are still emerging (See paragraph on Clinical description).

## Unresolved questions

Whilst the genetic basis of the X-linked form of CNM ("myotubular myopathy") has been known for a long time, recent years have seen the genetic resolution of a proportion of the autosomal-dominant and the autosomal-recessive forms of the condition. Despite these genetic advances, a substantial proportion of CNM cases remain currently still genetically unresolved, suggesting the existence of further gene loci. The pathological mechanisms leading to the skeletal muscle defects are still not understood and, although abnormal positioning of the nuclei is the histopathological hallmark of all genetically defined forms of CNM, the link between the mutated proteins and abnormal nuclear positioning remains unknown. Finally, considering that those concern ubiquitously expressed proteins, the tissue-specific expression of some of the implicated CNM mutations remains unaccounted for. Animal models may provide the basis for an advanced understanding of CNM and for future rational therapies of the condition.

## Competing interests

The authors declare that they have no competing interests.

## Authors' contributions

The authors equally contributed to this review article. They read and approved the final version of the manuscript.
